# Two Point Mutations on CYP51 Combined With Induced Expression of the Target Gene Appeared to Mediate Pyrisoxazole Resistance in *Botrytis cinerea*

**DOI:** 10.3389/fmicb.2020.01396

**Published:** 2020-06-30

**Authors:** Can Zhang, Muhammad Imran, Min Liu, Zhiwen Li, Huige Gao, Hongxia Duan, Shunli Zhou, Xili Liu

**Affiliations:** ^1^China Agricultural University, Beijing, China; ^2^State Key Laboratory of Crop Stress Biology for Arid Areas, Northwest A&F University, Yangling, China; ^3^Institute for the Control of Agrochemicals of Shaanxi Province, Xi’an, China

**Keywords:** *Botrytis cinerea*, pyrisoxazole, DMI, resistance, CYP51, point mutation, induced expression

## Abstract

*Botrytis cinerea* is a destructive plant pathogenic ascomycete that causes serious pre- and post-harvest losses worldwide. The novel sterol 14α-demethylase inhibitor (DMI) pyrisoxazole was recently registered for the control of tomato gray mold caused by *B. cinerea* in China. Baseline sensitivity of 110 *B. cinerea* isolates collected from nine provinces in China to pyrisoxazole was demonstrated, with a mean EC_50_ of 0.057 ± 0.029 μg/ml. Eleven stable mutants resistant to pyrisoxazole were generated via UV irradiation (RU-mutants) and spontaneous selection (RS-mutants) of conidia. The efficacy of pyrisoxazole against the resistant mutants was significantly lower than that of the sensitive isolates. Most of the pyrisoxazole- resistant mutants were less fit than the sensitive isolates, with reduced sporulation, conidia germination, sclerotium production, and pathogenicity, which was confirmed by the competitive ability test. Positive cross-resistance was only observed between pyrisoxazole and the DMIs tebuconazole and prochloraz, but not between pyrisoxazole and non-DMIs iprodione, procymidone, diethofencarb, fluazinam, pyrimethanil, or fludioxonil. A two-point mutation, at G476S and K104E in the RU-mutants, and a one point mutation, M231T, in the RS-mutants, were detected in the CYP51 protein of the resistant mutants. When exposed to pyrisoxazole, the induced expression level of *CYP51* increased in the resistant isolates as compared to sensitive ones. Molecular docking suggested that G476S and M231T mutations both led to the loss of electrostatic interactions between CYP51 and pyrisoxazole, while no change was found with the K104E mutation. Thus, two point mutations on CYP51 protein combined with induced expression of its target gene appeared to mediate the pyrisoxazole resistance of *B cinerea*.

## Introduction

Gray mold, caused by the ascomycete *Botrytis cinerea* Pers.: Fr. [teleomorph: *Botryotinia fuckeliana* (de Bary) Whetzel], is one of the most destructive diseases of crops both pre- and post-harvest (Williamson et al., 2007; Kumari et al., 2014). It causes considerable losses in over 200 types of economically valuable vegetables, fruits, and ornamental plants including tomato, cucumber, eggplant, zucchini, strawberry, and grape (Williamson et al., 2007; Samuel et al., 2011; Liu et al., 2016). Cultivation of plants in greenhouses increases the risk of infection by *B. cinerea* because the presence of high humidity is especially favorable to the pathogen (Rosslenbroich and Stuebler, 2000; Elad et al., 2007). In China, the percentage of yield loss caused by *B. cinerea* gray mold in vegetable production can be over 60% in some severely infected regions (Si et al., 2004a, b).

Control of gray mold is based on integrated management strategies, including cultivar resistance, physical factors, and inoculum reduction. Nevertheless, chemical control remains the main approach for the management of *B. cinerea* diseases (Leroux, 2007; Hahn, 2014; Fan et al., 2017; Yin et al., 2018). Several site-specific fungicides with different modes of action are available for gray mold management, including methyl benzimidazole carbamates (MBCs), anilinopyrimidines (APs), dicarboximides (DCFs), quinone outside inhibitors (QoIs), succinate dehydrogenase inhibitors (SDHIs), and phenylpyrroles (PPs). Unfortunately, according to the Fungicide Resistance Action Committee (FRAC)^1^, *B. cinerea* has been categorized as a high-risk pathogen due to the development of fungicide resistance. Over the last 30 years worldwide, the resistance of *B. cinerea* to these types of fungicides was frequently reported soon after their introduction for gray mold control (Moyano et al., 2004; Myresiotis et al., 2007; Zhang et al., 2009; Fernández-Ortuño et al., 2012; Chen et al., 2016; Yin et al., 2018). Resistance of *B. cinerea* often compromises the efficacy of fungicide and results in disease control failure. The most common disease management practice consists of alternation or tank mixtures of fungicides with different modes of action.

Sterol 14α-demethylase inhibitors (DMIs) are a major group of systemic fungicides that exhibit broad spectra of antifungal activity. The continued and successful use of DMI fungicides, even with the emergence of resistance, justifies the development of new fungicides in this class (Chen et al., 2012). The novel fungicide pyrisoxazole (previous Development Code No. SYP-Z048), 3-[5-(4-chlorophenyl)-2,3-dimethyl- 3-isoxazolidinyl] pyridine, is developed by the China Shenyang Sinochem Agrochemicals R&D Co., Ltd. (Former: China Shenyang Research Institute of Chemical Industry) (Supplementary Figure S1; Chen et al., 2015). This fungicide belongs to the pyridine subgroup within the DMI class, like pyrifenox that also has been shown in Supplementary Figure S1 (FRAC). Pyrisoxazole has shown great promise for the control of a broad range of fungi (Liu et al., 2004; Si et al., 2004a, b). It has been officially registered for the control of tomato gray mold in China under the tradename Junsiqi in 2008 (China Pesticide Information Network)^2^, and is currently being developed for applications on other target pathogens, such as against *Sclerotinia sclerotiorum* in oilseed rape (Duan et al., 2018b).

Until now, no systematic information has been available on the risk and molecular mechanism(s) of pyrisoxazole resistance in *B. cinerea*. Only one study has reported pyrisoxazole sensitivity in isolates sampled from the Liaoning Province in China (Zhu et al., 2016). Knowing the resistance basis of fungicides in pathogens is vital for disease management. The basis of resistance of DMI fungicides is most commonly conferred by (i) mutations in the *CYP51* gene, (ii) increased expression of the *CYP51* gene, or (iii) increased expression of membrane-bound transporters (Hayashi et al., 2001; Ma and Michailides, 2005; Kretschmer et al., 2009; Cools et al., 2013; Leroux and Walker, 2013). Pyrisoxazole resistance in *Monilinia fructicola* mutants was correlated with the presence of a mutation in the *CYP51* gene that caused an amino acid change from tyrosine to phenylalanine at position 136 (Chen et al., 2012, 2015).

The objectives of this study were to (i) establish a baseline sensitivity of *B. cinerea* isolates from China to the DMI fungicide pyrisoxazole, (ii) generate and characterize pyrisoxazole-resistant mutants to assess the risk of *B. cinerea* developing resistance to pyrisoxazole, and (iii) explore molecular mechanisms that might be responsible for resistance to pyrisoxazole. Such data are essential for subsequent fungicide resistance monitoring programs, as we found two point mutations G476S and M231T in the target protein CYP51 as well as induced expression of the target gene both appeared to contribute to the resistance of *B. cinerea* to pyrisoxazole.

## Materials and Methods

### Isolates and Culture Conditions

A total of 110 *B. cinerea* isolates were isolated from diseased tomato leaves or fruits between 2008 and 2012 from nine provinces in northern, central, and southern China where there was no history of pyrisoxazole usage (Supplementary Table S1). After purification by single-spore isolation, the isolates were firstly identified as *B. cinerea* and its closely related species by polymerase chain reaction (PCR) using a previously reported primer pair Bc-f/Bc-r designed on the basis of a sequence-characterized amplified region marker (Fan et al., 2015). Then, a PCR-restriction fragment length polymorphism (RFLP) analysis of the 18S/28S rRNA intergenic spacer (IGS) region with primers IGS1a and IGS1b was performed to distinguish *B. cinerea* and *B. pseudocinerea*, and the PCR product was digested with HinfI as previously described (Kretschmer and Hahn, 2008). All isolates were cultured in darkness on potato dextrose agar (PDA) plates at 20°C. For long-term storage, mycelia of each isolate were maintained in 5 ml plastic tubes containing PDA medium slants under mineral oil at 14°C.

### Sensitivity of *B. cinerea* Isolates to Pyrisoxazole

Pyrisoxazole was standard grade and kindly provided by China Shenyang Sinochem Agrochemicals R&D Co., Ltd. The pyrisoxazole sensitivity of 110 B. cinerea single-spore field isolates was determined by calculating the 50% inhibitory dose (EC_50_ value) on fungicide-amended PDA in Petri plates according to the method previously described in Cai et al. (2015). The fungicide concentrations are shown in Supplementary Table S2. The final concentration of DMSO solvent in the medium was 0.1%. After incubation for 3 days in darkness at 20°C, each colony diameter (minus 5 mm for the inoculated plug) was measured in two perpendicular directions. Each combination of isolate and fungicide was represented by three replicate plates, and the experiment was performed twice.

### Generation of Pyrisoxazole-Resistant Mutants

Ultraviolet (UV) irradiation and spontaneous selection of *B. cinerea* mycelium and conidia on pyrisoxazole-amended media were used in attempts to generate mutants. For the generation of UV irradiation mutants from mycelium, Petri dishes containing 3-day-old actively growing colonies of four DMI-sensitive isolates with different geographical origins and good fitness (S11, H9, T39, NJ10) were exposed for 40-50 min to a 254 nm UV light source placed 10 cm above the cultures. The exposed colonies were incubated in darkness for 1 h, after which mycelial plugs (5 mm in diameter) were excised from the dishes and transferred to PDA plates amended with pyrisoxazole at 1.0 μg/ml (minimal inhibitory concentration). After incubation at 20°C for 7–10 days in darkness, actively growing colonies were transferred to fresh fungicide-free PDA. Five days later, mycelial plugs from the colony periphery were transferred to PDA medium amended with pyrisoxazole at 1.0 μg/ml (the discriminatory concentration) to confirm resistance.

To induce conidia production, mycelial plugs (5 mm) excised from the margin of a 3-day-old PDA colony were placed upside-down on carrot agar medium (CA; 200 g of carrot, 15 g of agar, and distilled water to 1 L). After a 14 h of light and 10 h of darkness for 5-7 days, the conidia were harvested, suspended in 1 ml of sterile water, then treated with UV light (20 W, 254 nm) at 10 cm vertical distance for 45 s at 20°C, followed by 1 h of incubation in darkness to avoid light repair of DNA damage. The conidia were evenly distributed on CA medium amended with pyrisoxazole at 1.0 μg/ml. After 10 days of incubation, developing colonies were transferred to fresh fungicide-free PDA and then transferred to PDA medium amended with pyrisoxazole at 1.0 μg/ml to confirm resistance.

For the generation of spontaneous mutants, the same procedure as above was used but without UV irradiation. The determination of EC_50_ values for putative mutants was conducted as described previously. Each isolate was tested in triplicate and the experiment was repeated once. The mutation frequency as a percentage was calculated as the number of mutants/total number of treated conidia or mycelial plugs × 100; the resistance factor (RF) was calculated according to the formula EC_50_ value of the mutant/ EC_50_ value of its sensitive parent.

### Investigation of Control Efficacy Using Detached Leaf Assays

Four isolates, including two sensitive parental isolates (S11 and NJ10) and two resistant isolates (RU11-7 and RS10-1), were used to assess fungicide performance on treated leaves, as described previously (Zhang et al., 2019). Briefly, commercial-variety tomato (cv. BeiBei) leaves of similar size with no fungicide application were rinsed with water for 1 min, and air-dried. The detached leaves were sprayed to run-off with two concentrations (50 or 100 μg/ml) near the label rate of pyrisoxazole (75–150 μg/ml) diluted in Tween-80 water, respectively. Control leaves were sprayed with sterile distilled Tween-80 water. Leaves were allowed to dry overnight at 20°C. Mycelial plugs (5 mm in diameter) taken from the margins of the actively growing colony were placed upside down on the leaves. After inoculation, the plastic boxes were sealed with plastic bags to keep the relative humidity near 100%. After 5 days at 20°C with 12 h of light and 12 h of darkness, the lesion area on each leaf was measured. The experiment was conducted twice independently. Control efficacy was calculated according to the following formula: Control efficacy = ([lesion area with water application – lesion area with fungicide treatment] / lesion area with water application) × 100%.

### Stability of Pyrisoxazole Resistance in Mutants

Mycelial plugs taken from the periphery of actively growing colonies were transferred a total of 10 times to fresh, fungicide-free PDA medium. EC_50_ values for pyrisoxazole were determined before the transfer and after the 5th and 10th transfer. Each mutant was evaluated in triplicate, and the entire experiment was conducted twice.

### Mycelial Growth Rate Over a Series of Temperatures

Mycelial plugs (5 mm in diameter) of each isolate were transferred from the leading edge of an actively growing colony to PDA dishes and incubated in darkness at 4, 20, 25, 28, or 39°C. Colony diameter was measured after 2 days of incubation. Each combination of isolate or mutant and temperature was represented by three replicate plates, and the experiment was conducted twice.

### Conidia Production and Germination Rate *in vitro*

Conidia were induced as described in the previous section and harvested by rinsing the sporulating colony in each plate with 10 ml of a 0.1% aqueous glucose solution. The number of conidia in the suspension were counted with a hemacytometer and a microscope. To determine the rate of germination, the conidial suspension was adjusted to 10^5^-10^6^ conidia/ml and 50 μl of the suspension was spread onto depression slides placed on top of wet filter paper in a petri dish. The depression slides were incubated at 20°C for 6 h in darkness. In total, 200 conidia were assessed per isolate for germination ability under a microscope. Each isolate was tested in triplicate and the entire experiment was repeated once.

### Pathogenicity, Conidial Production, and Germination Rate *in vivo*

Pathogenicity of *B. cinerea* was determined on detached leaves of “BeiBei” as described above, but without fungicide treatment. Controls consisted of leaves that were punctured and then inoculated with a sterile agar plug or distilled water. After 5 days at 20°C with 12 h of light and 12 h of darkness, the lesion area on each leaf was measured. The experiments were performed twice.

Conidia production *in vivo* was measured by inoculating leaves as described in the previous paragraph. After incubation at 20°C for another 7–10 days, the number of conidia and the germination rate were determined in a 0.1% aqueous glucose solution. Each isolate was tested in triplicate and the *in vivo* sporulation experiments were performed twice.

### Sclerotia Production

For comparison of sclerotia production, an agar plug cut from the edge of a 3-day-old colony on PDA was placed in the center of a 9 cm diameter Petri dish. After 20 days at 20°C in darkness, sclerotia were removed from the cultures and dried to a constant weight at 50°C for 16 h. Sclerotia production was expressed as sclerotia dry mass per Petri dish. Each isolate or mutant was represented by three replicate Petri dishes, and the experiment was conducted twice.

### Competitive Ability

Competitive ability was determined for five combinations of the sensitive parental isolates (S) and their resistant mutants (R): S11 versus RU11-7, S11 versus RU11-21, NJ10 versus RS10-1, NJ10 versus RS10-4, and NJ10 versus RS10-5. Each combination consisted of three ratios of the S and R isolates (9R:1S, 5R:5S, and 1R:9S), which were prepared by mixing conidial suspensions of the pyrisoxazole sensitive isolates and the corresponding resistant mutants. Conidial suspensions were prepared as described earlier, and the final concentration for each was adjusted to 1 × 10^5^/ml with the aid of a hemacytometer and microscope. A 100 μl volume of each of the resulting conidial suspensions was spread on a CA plate, and the cultures were grown, and conidia were harvested as described earlier. This procedure was repeated five times (five subcultures). After the 1st, 3rd, and 5th subculture, 70 single conidial isolates were obtained and transferred to PDA plates amended with 1 μg/ml pyrisoxazole. Isolates that were able to grow on the plates amended with the discriminatory concentration of pyrisoxazole were considered resistant, and those that could not were considered sensitive. The ratios of the resistant and sensitive isolates obtained after subculture on CA were calculated and compared with the original ratios. This experiment was performed twice.

### Cross-Resistance

The pyrisoxazole-resistant mutants and their parents were exposed to two DMIs (tebuconazole, and prochloraz) belonging to different chemical groups, and five other fungicides of different modes of action (iprodione, procymidone, diethofencarb, fluazinam, pyrimethanil, and fludioxonil). The EC_50_ values for each isolate/fungicide combination were determined using the aforementioned mycelial growth inhibition method, except that glucose-gelatin-agar medium (0.4% glucose, 0.4% gelatin, 0.177% K_2_HPO_4_, 0.036% MgSO_4_⋅7H_2_O, and 1.2% agar) was used for pyrimethanil (Birchmore et al., 1996), and salicylhydroxamic acid was added to the azoxystrobin-amended medium with a final concentration of 100 μg/ml to inhibit the alternative respiration pathway (Bardas et al., 2010). The concentrations used for each fungicide are provided in Supplementary Table S2. Each treatment had three replicate plates, and the experiment was conducted twice.

### Cloning and Sequencing of the *BcCYP51* Gene

Genomic DNA was extracted from mycelium, as described previously (Chi et al., 2009). The complete *BcCYP51* gene was amplified with primer pair BcCYP51-F and BcCYP51-R as described previously (Albertini et al., 2002). The amplified *BcCYP51* full-length fragments were purified using the EasyPure Quick Gel Extraction Kit (TransGen, Beijing, China) and were cloned into the pEASY-T1 simple plasmid (TransGen, Beijing, China) according to the manufacturer’s recommendations. The vector inserted into *Escherichia coli* was sequenced (Sunbiotech Co., Beijing) using vector primers M13F (5′-ACTGGCCGTCGTTTTAC-3′) and M13R (5′-GTCCTTTGTCGATACTG-3′). DNA sequences were analyzed with DNAMAN software (Lynnon BioSoft, Quebec, Canada).

### Determination of the Expression Level of *CYP51*

Each isolate was grown in 150 ml Erlenmeyer flasks containing 80 ml of PDB liquid medium. After 3 days at 20°C on a rotary shaker at 150 rpm, pyrisoxazole (EC_50_ value for each isolate) was added to three of the six flasks for each isolate. After 24 h, mycelia were harvested by vacuum filtration for RNA extraction. Total RNA was extracted by the SV Total RNA Isolation Kit (Promega, Beijing, China), and cDNA was synthesized using the PrimeScript RT Reagent Kit with gDNA Eraser (Takara, Beijing, China), following the manufacturer’s instructions.

The expression level of the target genes was quantified by quantitative real-time PCR (qRT-PCR), which was performed with an ABI7500 sequence detection system (Applied Biosystems, United States). qRT-PCR was conducted in a 20 μl reaction volume with the SYBR Premix Dimer Eraser Kit (Takara, Beijing, China). The following program were used for the qRT-PCR analysis by three biological replications: 95°C for 30 s, followed by 40 cycles of 95°C for 5 s, 60°C for 30 s, and 72°C for 34 s. Primers used for qRT-PCR were CYP51-QF/QR (5′-ATTTGGTGCTGGCAGACATAGA-3′; 5′-GTGAATAAACTTGCGTAATCG GTA-3′) for the *BcCYP51* gene and Actin-QF/QR (5′-CTGGTCGTGATTT GACTGATTA-3′; 5′-GATTGACTGGCGGT TTGG-3′) for the reference gene *actin*. The relative quantities (RQs) of products were calculated using the 2^–ΔΔCt^ method (Livak and Schmittgen, 2001). Three independent experiments were conducted.

### Molecular Docking Analysis

Bioinformatic analysis was used to investigate the molecular docking of pyrisoxazole with the BcCYP51 protein. The crystal structure of 5HS1, a CYP51 protein from the yeast *Saccharomyces cerevisiae* bound with voriconazole, was retrieved from the Protein Data Bank and used in the current study. Sequence alignment by DNAMAN software indicated that the CYP51 amino acid residues of *S. cerevisiae* showed about 45% sequence identity with that of *B. cinerea*. The crystal structure 5HS1 was a suitable template for studying the binding conformation of pyrisoxazole with the BcCYP51 protein.

The modeling structure of *B. cinerea* was obtained by using the template 5HS1 and the online Swiss-model software^3^. The binding cavity was predicted by a ligand mode for voriconazole complexed in CYP51 by the SYBYL 7.3 software program. The Biopolymer-Replace Sequence subset from the Sybyl X2.0 software package was used to produce site-directed mutations at residues K104, G476, and M231, respectively. The Tripos force field with Gasteiger–Marsili charges was used for energy minimization. The molecular conformations of pyrisoxazole were constructed by Sketch mode and were optimized using the Tripos force field and Gasteiger–Hückel charge. The Surflex-Dock of SYBYL 7.3 was used for the molecular docking modeling (Jain, 2003). The binding cavity was set as “ligand,” and the total score was used to evaluate the binding affinity between ligand and protein (Jain, 1996). All molecular modeling between the putative *B. cinerea* CYP51 proteins with ligand was conducted on the Silicon Graphics (SGI) Fuel Workstation (Silicon Graphics International Corp., CA, United States).

### Statistical Analysis

Data were analyzed by SPSS software (ver. 21.0) based on an unpaired Student’s *t-*test. An asterisk indicates significant differences with the *P-*values marked (^∗^*P* < 0.05; ^∗∗^*P* < 0.01, ^∗∗∗^*P* < 0.001). Cross-resistance between two fungicides was analyzed using Spearman’s rank correlation coefficient with log-transformed EC_50_ values.

## Results

### Baseline Sensitivity of *B. cinerea* in China to Pyrisoxazole

**FIGURE 1:**
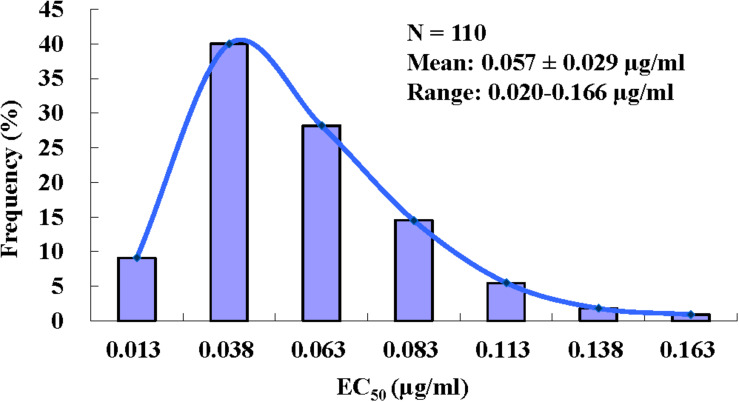
Frequency distribution of effective concentrations for 50% growth inhibition (EC_50_) of pyrisoxazole against 110 *Botrytis cinerea* isolates.

The EC_50_ values required for pyrisoxazole to inhibit mycelial growth of *B. cinerea* field isolates collected from nine provinces in China ranged from 0.020 to 0.166 μg/ml, with a mean EC_50_ of 0.057 ± 0.029 μg/ml (Figure 1). The ratio of the maximum EC_50_ to the minimum EC_50_ was 8.3. The range and mean of EC_50_ values in different populations are listed in Supplementary Table S1. The narrow range and low EC_50_ values of the *B. cinerea* isolates indicate no preexisting pyrisoxazole resistance.

### Generation of Pyrisoxazole-Resistant Mutants

No mutants were obtained from UV irradiation and spontaneous selection of mycelium. Six resistant mutants, designated as RU11-1, RU11-5, RU11-7, RU11-12, RU11-15, and RU11-21, were obtained from conidia of parental wild-type isolate S11 treated with UV light. Five mutants, designated as RS10-1 to RS10-5, were obtained from spontaneous selection from the conidia of parental isolate NJ10. EC_50_ values of the mutants from UV irradiation ranged from 0.293 to 0.565 μg/ml, with corresponding resistance factors of 4.7–9.1 (Table 1). The mutation frequency from UV irradiation of conidia was 6.5 × 10^–9^. EC_50_ values of the mutants from spontaneous selection of conidia ranged from 0.337 to 0.453 μg/ml with corresponding resistance factors of 7.6–10.1 (Table 1). The average mutation frequency from spontaneous selection of conidia was 5.0 × 10^–9^.

**TABLE 1:** Stability and level of pyrisoxazole resistance of two groups of *Botrytis cinerea* mutants and their parental isolates, S11 and NJ10, after the 1st, 5th, and 10th subculture on fungicide-free medium.

Isolate^a^	EC_50_ (μg/ml)			RF^b^			FSC^c^
	1st	5th	10th	1st	5th	10th	
S11	0.062	0.055	0.057	–	–	–	–
RU11-3	0.545	0.375	0.465	8.8	6.8	8.2	0.9
RU11-5	0.310	0.268	0.350	5.0	4.9	6.1	1.2
RU11-7	0.293	0.229	0.342	4.7	4.2	6.0	1.3
RU11-12	0.326	0.313	0.353	5.3	5.7	6.2	1.2
RU11-15	0.438	0.313	0.374	7.1	5.7	6.6	0.9
RU11-21	0.565	0.455	0.417	9.1	8.3	7.3	0.8

NJ10	0.045	0.049	0.046	–	–	–	–
RS10-1	0.337	0.316	0.355	7.6	6.4	7.7	1.0
RS10-2	0.401	0.379	0.331	8.9	7.7	7.2	0.8
RS10-3	0.385	0.418	0.417	8.6	8.5	9.1	1.1
RS10-4	0.358	0.327	0.327	8.0	6.7	7.1	0.9
RS10-5	0.453	0.440	0.433	10.1	9.0	9.4	0.9

*^a^S11 and NJ10 correspond to the wild-type parental isolates. ^b^RF = resistance factor, the radio of the EC_50_ of the fungicide-resistant mutant to that of its wild-type parent. ^c^FSC = factor of sensitivity change, the radio of RF values for EC_50_ at the 10th to the 1st transfer.*

### Detached Leaf Assays

**FIGURE 2:**
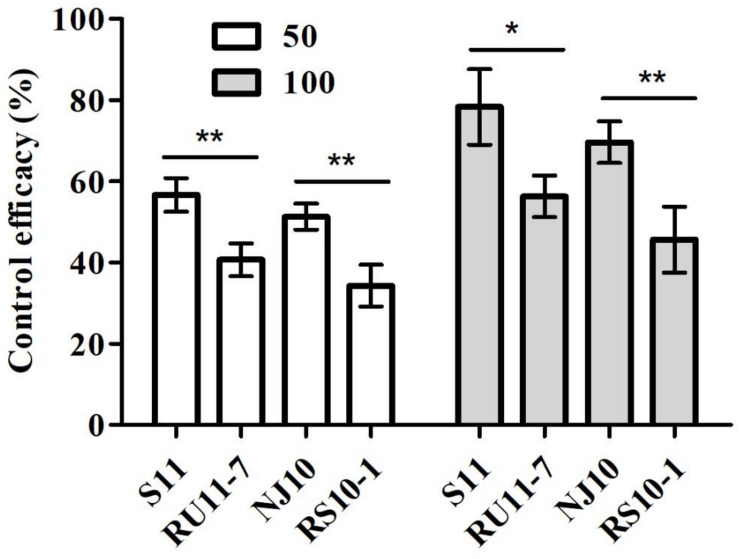
Control efficacy of gray mold of pyrisoxazole-treated tomato leaf inoculated with pyrisoxazole-sensitive (S11, NJ10) and resistant (RU11-7, RS10-1) isolates. Columns and bars indicate means ± standard deviation (SD) from replicates. An asterisk indicates significant differences based on an unpaired Student’s *t-*test with the *P-*values marked (*P < 0.05; **P < 0.01, ***P < 0.001).

All isolates were able to infect and develop lesions on tomato leaves without fungicide treatment. Lesion development of DMI-sensitive isolates (S11 and NJ10) or resistant isolates (RU11-7 and RS10-1) was decreased on leaves sprayed with pyrisoxazole, compared with the control treatment. However, the control efficacy for two resistant isolates with the lowest resistance factors was about 20% lower than that for the sensitive isolates at the two tested fungicide concentrations (Figure 2). The results demonstrated that there was a significant difference (*P* < 0.05) in control efficacy between sensitive and resistant isolates, indicating that reduced sensitivity of *B. cinerea* isolates to pyrisoxazole did occur.

### Resistance Level and Stability

To determine the fitness of pyrisoxazole-resistant mutants, the level of pyrisoxazole resistance of the mutants was measured, and the RF values (resistance factor, the ratio of the EC_50_ value of a resistant mutant to that of its parent) was determined. After 10 transfers on fungicide-free PDA, the FSC values (factor of sensitivity change, the ratio of RF values at the 10th to the 1st transfer) ranged from 0.8 to 1.3, indicating that resistance was stable.

### Colony Growth as Affected by Temperature

For all of the pyrisoxazole-resistant mutants and their parental isolate of *B. cinerea*, the optimal temperature for mycelial growth was 20°C (Table 2). Most mutants grew at the same rate as their parental isolates at all the tested temperatures, except RU11-3 and RU11-21: these grew slower than their parental isolate S11 at 20, 25, and 28°C (Table 2). All isolates of *B. cinerea* did not grow at 39°C.

**TABLE 2:** Effect of temperature on the mycelial growth of resistant mutants of *Botrytis cinerea* and their wild-type parents on PDA without fungicide.

Isolate^a^	Colony diameter (mm)^b^				
	4°C	20°C	25°C	28°C	39°C^c^
S11	0.6 ± 0.1	4.1 ± 0.1	3.8 ± 0.2	2.1 ± 0.1	–
RU11-3	0.6 ± 0.1	3.7 ± 0.2*	3.1 ± 0.2**	1.6 ± 0.1**	–
RU11-5	0.4 ± 0.0	4.2 ± 0.1	3.7 ± 0.2	1.8 ± 0.1	–
RU11-7	0.4 ± 0.1	4.0 ± 0.1	3.1 ± 0.1*	1.8 ± 0.2	–
RU11-12	0.5 ± 0.0	4.2 ± 0.1	3.8 ± 0.2	1.9 ± 0.2	–
RU11-15	0.4 ± 0.1	4.0 ± 0.1	4.0 ± 0.2	1.8 ± 0.1	–
RU11-21	0.3 ± 0.0*	3.5 ± 0.2**	3.1 ± 0.2**	1.5 ± 0.1**	–

NJ10	0.6 ± 0.0	4.2 ± 0.1	3.6 ± 0.2	2.2 ± 0.1	–
RS10-1	0.4 ± 0.1	4.2 ± 0.2	3.5 ± 0.2	1.9 ± 0.1	–
RS10-2	0.4 ± 0.0	4.2 ± 0.1	3.7 ± 0.2	1.9 ± 0.1	–
RS10-3	0.5 ± 0.1	4.0 ± 0.1	3.4 ± 0.3	1.8 ± 0.2	–
RS10-4	0.4 ± 0.0	4.1 ± 0.1	3.7 ± 0.2	1.9 ± 0.2	–
RS10-5	0.4 ± 0.1	4.0 ± 0.2	3.7 ± 0.3	1.8 ± 0.1	–

*^a^Colony diameter was measured after incubation for three days at 20°C. S11 and NJ10 correspond to the wild-type parental isolates. ^b^Values in a column indicate means ± SD. An asterisk indicates significant differences based on an unpaired Student’s t-test with the P-values marked (^∗^P < 0.05; ^∗∗^P < 0.01, ^∗∗∗^P < 0.001). ^c^“–”means the isolates could not grow at 39°C.*

### Most Resistant Mutants Experienced Fitness Penalty

The average mycelial growth rate did not significantly differ for most pyrisoxazole-resistant mutants compared to the parental wild-type isolate, except for RU11-3 and RU11-21 (Table 3). Spore production was significantly lower for the mutants than for their parents except for RU11-21 and RS10-1, regardless of whether produced *in vitro* or *in vivo* (Table 3). All the RU-mutants had a reduced conidia germination rate, and the RS-mutants exhibited similar germination rates to that of the wild-type isolate in *vivo* (Table 3). Sclerotium production was significantly reduced in the RU-mutants but was higher in the RS-mutants except for RS10-1 (Table 3). The lesion area produced on detached tomato leaf was significantly reduced in the mutants compared to the parental wild-type isolate (Table 3).

**TABLE 3:** Comparison of fitness parameters for resistant *Botrytis cinerea* mutants and their wild-type parental isolate^a^.

		*In vitro*		*In vivo*			
Isolate^b^	Mycelial growth (cm/day)^c^	Sporulation (×10^5^/cm^2^)	Germination rate (%)	Sporulation (×10^5^/cm^2^)	Germination rate (%)	Sclerotia production (g/Petri dish)	Lesion area (mm^2^)
S11	2.1 ± 0.1	5.3 ± 0.7	85.4 ± 2.1	9.7 ± 1.1	96.3 ± 1.7	0.045 ± 0.006	2.9 ± 0.3
RU11-3	1.8 ± 0.2*	1.2 ± 0.1***	38.7 ± 3.4***	7.3 ± 0.3***	48.4 ± 2.1***	0.135 ± 0.012***	1.7 ± 0.3***
RU11-5	2.1 ± 0.1	0.7 ± 0.1***	0.7 ± 0.3***	5.5 ± 0.3***	61.0 ± 1.7***	0.176 ± 0.003***	2.2 ± 0.2***
RU11-7	2.0 ± 0.1	0.8 ± 0.4***	3.3 ± 0.6***	0.4 ± 0.1***	8.2 ± 2.4***	0.140 ± 0.006***	1.9 ± 0.2***
RU11-12	2.1 ± 0.1	0.8 ± 0.2***	20.5 ± 2.4***	4.9 ± 0.7***	33.3 ± 1.0***	0.173 ± 0.008***	2.2 ± 0.2***
RU11-15	2.0 ± 0.1	1.5 ± 0.2***	57.1 ± 1.6***	8.7 ± 0.3**	57.2 ± 2.6***	0.155 ± 0.024***	2.0 ± 0.2***
RU11-21	1.8 ± 0.2**	5.2 ± 0.8	34.3 ± 3.2***	9.2 ± 0.3	23.8 ± 3.3***	0.078 ± 0.007**	1.6 ± 0.1***

NJ10	2.1 ± 0.1	5.2 ± 0.7	54.2 ± 5.9	8.6 ± 0.9	90.6 ± 3.0	0.257 ± 0.032	2.2 ± 0.4
RS10-1	2.1 ± 0.2	5.2 ± 0.2	78.3 ± 4.1**	8.3 ± 0.3	92.4 ± 2.1	0.213 ± 0.029	0.3 ± 0.1***
RS10-2	2.1 ± 0.1	1.2 ± 0.4***	60.6 ± 1.2	2.5 ± 0.6***	88.5 ± 1.7	0.201 ± 0.022*	0.7 ± 0.3***
RS10-3	2.0 ± 0.1	2.3 ± 0.7***	45.8 ± 9.3	0.9 ± 1.0***	90.2 ± 2.4	0.196 ± 0.019*	1.1 ± 0.2***
RS10-4	2.1 ± 0.1	2.0 ± 0.2***	70.1 ± 3.7**	3.6 ± 0.9***	93.4 ± 1.8	0.002 ± 0.001***	1.4 ± 0.2***
RS10-5	2.0 ± 0.2	0.9 ± 0.2***	72.0 ± 6.0**	2.7 ± 1.3***	87.2 ± 5.6	0.113 ± 0.021***	1.6 ± 0.3***

*^a^Values in a column indicate means ± standard deviation (SD). An asterisk indicates significant differences based on an unpaired Student’s t-test with the P-values marked (^∗^P < 0.05; ^∗∗^P < 0.01, ^∗∗∗^P < 0.001). ^b^S11 and NJ10 correspond to the wild-type parental isolates. ^c^Growth rate was determined at the optimum temperature of 20°C.*

### Competitive Ability

**FIGURE 3:**
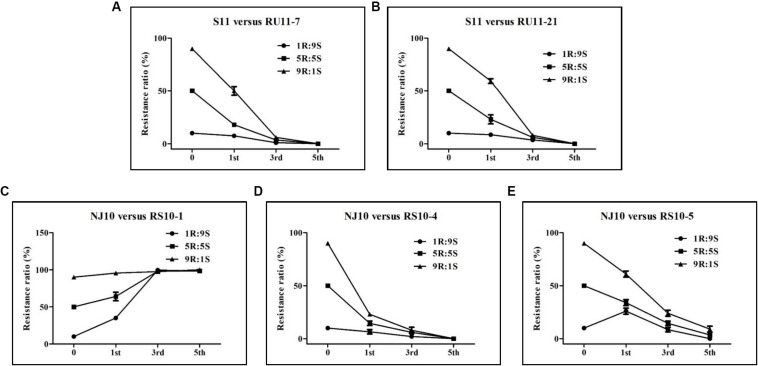
Competition between pyrisoxazole-sensitive and resistant isolates of *Botrytis cinerea* as indicated by the resistance ratios after 1st, 3rd, and 5th subcultures on agar medium. Five combinations of the sensitive parental isolates (S) and their resistant mutants (R) were determined: **(A–E)** representing S11 versus RU11-7, S11 versus RU11-21, NJ10 versus RS10-1, NJ10 versus RS10-4, and NJ10 versus RS10-5, respectively. Each combination consisted of three ratios of the S and R isolates (9R:1S, 5R:5S, and 1R:9S), which were prepared by mixing conidia suspensions of the pyrisoxazole-sensitive isolates and the corresponding resistant mutants.

The observed frequency of pyrisoxazole-resistant isolates was less than the expected frequency in the combinations S11 versus RU11-7, S11 versus RU11-21, and NJ10 versus RS10-4 regardless of whether it was after the 1st, 3rd, or the 5th transfers (Figures 3A,B,D). For the NJ10 versus RS10-5 combination, the drop in the observed frequency of pyrisoxazole-resistant isolates occurred after the 3rd transfer (Figure 3E). However, RS10-1 showed a higher competitive ability than its parent NJ10 as indicated in Figure 3C. The results indicated that most of the pyrisoxazole-resistant isolates were less fit than the sensitive wild-type isolates, except RS10-1.

### Cross-Resistance

**FIGURE 4:**
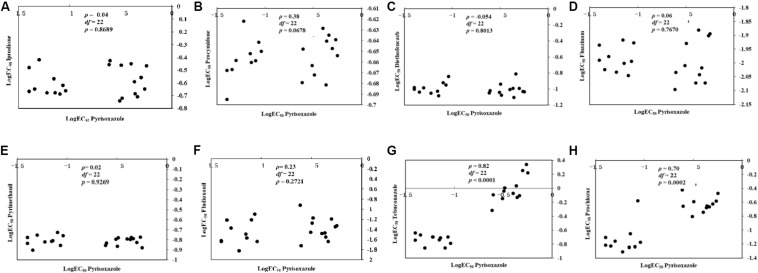
Cross-resistance between pyrisoxazole and **(A)** iprodione, **(B)** procymidone, **(C)** diethofencarb, **(D)** fluazinam, **(E)** pyrimethanil, **(F)** fludioxonil, **(G)** tebuconazole, or **(H)** prochloraz by rank correlation analysis. Data shown in logarithmic values of effective concentrations for 50% mycelial growth inhibition (log EC_50_) among *Botrytis cinerea* for fungicide combinations.

For the 11 pyrisoxazole-resistant mutants and 11 sensitive field isolates tested, there was no correlation between sensitivity to pyrisoxazole and that to iprodione, procymidone, diethofencarb, fluazinam, pyrimethanil, or fludioxonil (Figures 4A–F). A positive correlation was observed between sensitivity to pyrisoxazole and that to DMIs tebuconazole (ρ = 0.82, *P* < 0.0001) and prochloraz (ρ = 0.70, *P* = 0.0002) (Figures 4G,H).

### Molecular Analysis of the CYP51 Gene in *B. cinerea*

**FIGURE 5:**
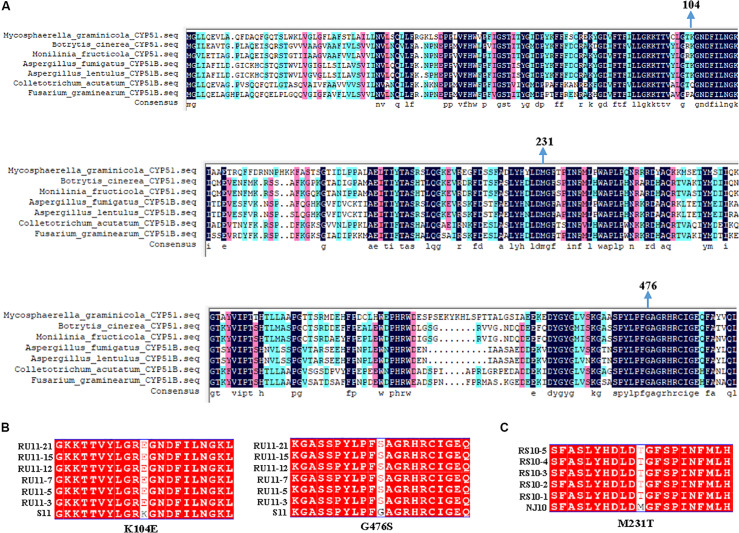
Multiple sequence alignment of the CYP51 proteins in *Botrytis cinerea* sensitive parental isolates with that in **(A)** six other fungal species; **(B)** six resistant mutants (RU-mutants) generated from UV irradiation of conidia; **(C)** five resistant mutants (RS-mutants) acquired from the spontaneous selection of conidia. S11 and NJ10 correspond to the wild-type parental isolates.

**FIGURE 6:**
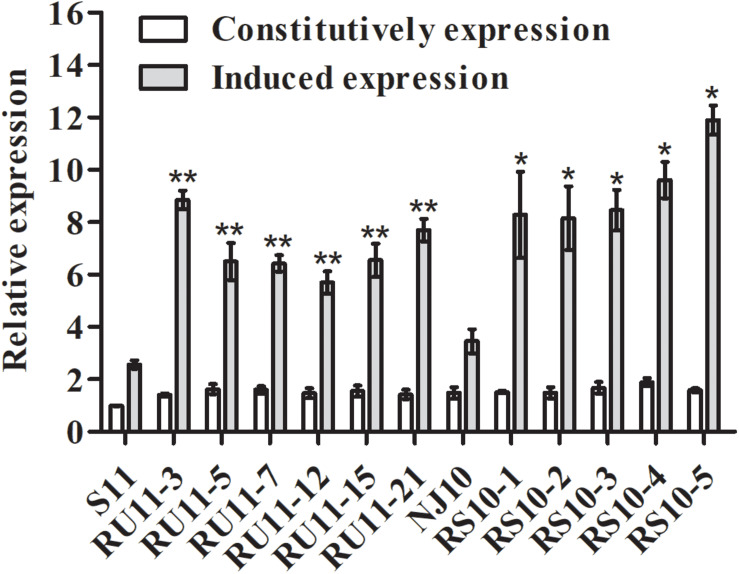
Constitutive and fungicide-induced expression of *CYP51* from pyrisoxazole resistant *Botrytis cinerea* mutants and the corresponding sensitive parental isolates S11 and NJ10. Relative expression of *CYP51* was calculated by the 2^–ΔΔCt^ method with the *actin* gene as reference. Expression in each isolate was relative to the expression of isolate S11. Columns and bars indicate mean ± SD for replicates. An asterisk indicates significant differences between each pyrisoxazole-resistant isolate and its parental sensitive isolate based on an unpaired Student’s *t-*test with the *P-*values marked (*P < 0.05; **P < 0.01).

The *CYP51* genes of the pyrisoxazole-resistant mutants and their parental isolates were cloned and sequenced (GenBank accession numbers: MT430873 for the sensitive isolate S11, MT430874 for the RU-mutant, MT430875 for the sensitive isolate NJ10, and MT430876 for the RS-mutant). An annotated alignment of CYP51 protein for several fungal species was shown in Figure 5A, and the ZtCYP51 sequence from *Zymoseptoria tritici* (accession number AY253234) was used as the archetype protein to unify the labeling of amino acids in fungicide target proteins (Figure 5A; Mair et al., 2016). Multiple sequence alignment revealed a two-point mutation, at amino acid positions 104 and 476 in all RU-mutants (Figure 5B), and a point mutation at amino acid position 231 in RS-mutants (Figure 5C). For the resistant mutants acquired from UV irradiation, lysine was substituted for glutamic acid at codon 104 (K104E), and glycine was substituted for serine at codon 476 (G476S). Methionine was substituted for threonine at codon 231 (M231T) for the mutants acquired from spontaneous selection (Figures 5B,C). The expression level of *CYP51* gene was further determined, with and without pyrisoxazole treatment. It is found that the expression level of *CYP51* was significantly induced by pyrisoxazole in the resistant mutants. It increased by 1.5–2.4-fold in RU-mutants compared to its parental wild-type isolate S11; and 2.2–3.2-fold in RS-mutants compared to NJ10 (Figure 6).

### Analysis of the Affinity Between the Mutation Positions and Pyrisoxazole

**FIGURE 7:**
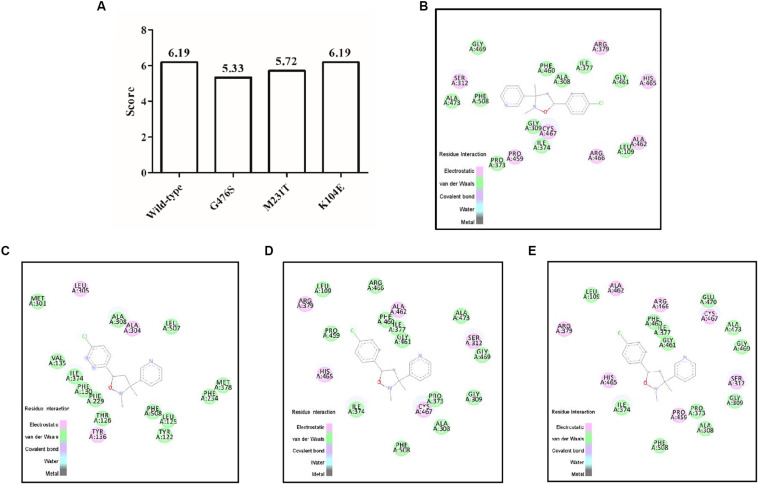
Docking of pyrisoxazole in the modeled binding pockets of CYP51 in *Botrytis cinerea*. The structure of *B. cinerea* was modeled using the template of 5HS1 from *Saccharomyces cerevisiae* by the online Swiss-model software. **(A)** Total scores of pyrisoxazole docked into the CYP51 binding site of *B. cinerea* wild-type and mutated models; **(B–E)** represent the binding pockets of pyrisoxazole in the wild-type CYP51 **(B)**, or with mutation G476S **(C)**, M231T **(D)**, or K104E **(E)** of *B. cinerea.*

Models were built for pyrisoxazole docking into the CYP51 binding pocket with the following mutations: K104E, G476S, and M231T. The docking scores of these predicted models are shown in Figure 7A. The model for the wild-type indicated that there were seven electrostatic interactions between CYP51 and pyrisoxazole (Figure 7B). The amino acid mutation G476S caused the migration of pyrisoxazole binding sites in the CYP51 and thereby led to the loss of four electrostatic interactions (Figure 7C). The docking score decreased from approximately 6.19–5.33 for this G476S mutation (Figure 7A). Two electrostatic interactions disappeared with the M231T mutation (Figure 7D). As a result, the docking score changed from 6.19 for M231 and to 5.72 for T231 (Figure 7A). No change was found with the K104E mutation, which is also reflected by the unchanged score (Figure 7E).

## Discussion

In this study, we established the baseline sensitivity of *B. cinerea* to pyrisoxazole using isolates from nine provinces in China, which represents the first step in monitoring and managing the development of fungicide resistance in the field (Russell, 2004). Given that the isolates were collected before pyrisoxazole was widely applied to fields in China, the EC_50_ values obtained provide a very accurate measure of the baseline sensitivity of *B. cinerea* to pyrisoxazole in this context. Zhu et al. (2016) determined the sensitivity of *B. cinerea* to pyrisoxazole from Liaoning province. As the isolates used in the present study were collected from the northern, central, and southern regions of China, the results can be used to provide a baseline for monitoring changes in the sensitivity of *B. cinerea* populations to pyrisoxazole in China. The low EC_50_ values (mean: 0.057 μg/ml) in this study were consistent with the previous report with the mean EC_50_ of 0.068 μg/ml (Zhu et al., 2016), and these provide strong evidence of no pyrisoxazole-resistant subpopulations existing in wild populations of *B. cinerea*. Moreover, the intrinsic activity of pyrisoxazole on *B. cinerea* was higher than most DMI fungicides, such as teuconazole used in the current study, as well as previous reported propiconazole and triflumizole (Markoglou and Ziogas, 2002; Song et al., 2016). In detail, the activity is about 5 or 10 times greater compared to teuconazole, or triflumizole, respectively. In addition, it showed a similar intrinsic activity with pyrifenox to *B. cinerea* (Hayashi et al., 2002). It is speculated that there may be some difference in the binding of DMIs in CYP51 protein of *B. cinerea*.

No mutants were obtained from mycelium of *B. cinerea*. The mutation frequency from UV irradiation and spontaneous selection of conidia was 6.5 × 10^–9^, and 5.0 × 10^–9^, respectively. This low mutation frequency is in contrast to a previous study reporting frequent development of *Z. tritici* mutants resistant to DMI fungicides via spontaneous selection at a relatively high rate of about 10^–4^ (Zwiers et al., 2002). Consistent with *B. cinerea*, no pyrisoxazole-resistant mutants were obtained by spontaneous selection in the study of *M. fructicola*, and the average mutation frequency from the conidia of *M. fructicola* exposed to UV irradiation under pyrisoxazole treatment was 8.3 × 10^–10^ (Chen et al., 2015). The very low frequency at which *B. cinerea* and *M. fructicola* generate pyrisoxazole-resistant mutants is mirrored by the fact that mutants have not been observed in fields sprayed with DMI fungicides multiple times or over several years. However, the average mutation frequency under pyrisoxazole treatment from the mycelium of *M. fructicola* was much higher, indicating that there is still the possibility to obtain mutants from *B. cinerea* mycelium. In the future work, more fungicide concentrations could be used for the screening of pyrisoxazole-resistant mutants.

On fungicide-treated tomato leaves, presumably the mutants with the lowest RF values are more difficult to control compared with sensitive isolates (Figure 2), indicating that reduced sensitivity of *B. cinerea* isolates to pyrisoxazole did occur. A comparison of the biological characteristics with the parental isolate indicated that there was a significant fitness penalty for most mutants, suggesting the spread of resistant mutants may be affected in the field. This was confirmed by the competitive ability test in which most of the resistant mutants showed a substantial drop in the observed frequency of pyrisoxazole-resistant isolates (Figure 3). However, some mutants bearing the same mutation in the target protein, such as RS10-1, showed an unimpaired fitness and even a higher competitive ability than its parent NJ10. It could be explained that some mutations might alter other areas of the genome as fitness penalty is not necessarily due to the mutations in the target CYP51 protein. Given the mutation frequency and cross-resistance results, the resistance risk of *B. cinerea* to pyrisoxazole may be considered to be low. Considering that the mutant RS10-1 still showed a good fitness and the control efficacy was clearly reduced when inoculating plants with these resistant mutants, it indicated that if such a mutation arises in field populations of *B. cinerea*, they could also possibly survive in the field. Therefore, on one hand, it is advisable to closely monitor any sensitivity changes to pyrisoxazole in *B. cinerea* populations in the field; on the other hand, more mutants with other phenotype and fitness should be obtained to further optimize this conclusion of resistance risk.

We found strong positive cross-resistance between pyrisoxazole and two DMIs in the mutants with different chemistries, and previous study also revealed cross-resistance between pyrisoxazole and propiconazole in *M. fructicola* mutants (Chen et al., 2015). Cross-resistance was not found between DMI and non-DMI fungicides in the current study. This is consistent with the cross-resistance reports on *M. fructicola* and *S. sclerotiorum* (Chen et al., 2015; Duan et al., 2018b). The efficacy of different commonly used fungicides has been decreased by the development of resistant *B. cinerea* isolates (as mentioned in “Introduction” section). Therefore, pyrisoxazole can be used in rotation for better management of gray mold. Mixtures of pyrisoxazole and the protective fungicide chlorothalonil or thiram have been reported to be highly effective for the control of *B. cinerea* (Shao et al., 2006). This can lower the selection pressure to slow down DMI resistance development.

Point mutations in the CYP51 protein and over-expression of the *CYP51* gene are two main determinants of DMI resistance. The third hypothesis is resulted from over-expression of transporter genes, which is usually indicated by different spectrum of cross-resistance in resistant mutants depend upon the transporter involved (Hayashi et al., 2001; Ma and Michailides, 2005). No cross-resistance was found between DMIs and six non-DMIs in the mutants, it indicated that increased expression of membrane-bound transporters was not the main reason for the fungicide resistance and more kinds of fungicides could be used to explain this issue in the future. In this study, we firstly cloned and characterized the target gene *CYP51* for *B. cinerea* and three point mutations K104E, G476S, M231T, were found in resistant *B. cinerea* mutants. Previous studies demonstrated that point mutation G461S in the MfCYP51 protein (corresponds to G476S in the reference ZtCYP51 sequence) is associated with tebuconazole resistance in *M. fructicola* populations in Brazil (Lichtemberg et al., 2017). Fujimura et al. (2016) listed the same mutation in *Podosphaera xanthii* resistant to DMI fungicides. Similar mutation was also reported in *Pyrenopeziza brassicae* with reduced tebuconazole sensitivity (Carter et al., 2014). This indicates these point mutations, especially the G476S mutation detected in the current study, might play roles in the resistance to pyrisoxazole of *B. cinerea*. Then, we found that the induced expression level of *CYP51* increased in the resistant isolates when exposed to pyrisoxazole (Figure 6). Therefore, it indicated that point mutations in the CYP51 protein and the increased expression of the *CYP51* gene both appeared to mediate the pyrisoxazole resistance of *B cinerea*.

The point mutations could change the conformation of the binding pocket in 14α-demethylase and reduce its potential to bind DMI fungicides (Sionov et al., 2012; Zhang et al., 2017; Qian et al., 2018). Molecular docking was used to explore the conformation change in the binding pocket of CYP51 of *B cinerea*, with or without point mutations. As expected, mutation G476S in CYP51 changed the conformation of pyrisoxazole in the binding pocket, and G476S and M231T mutations both led to the loss of electrostatic interactions, indicating these two mutations contributed to pyrisoxazole resistance the *B cinerea*. In addition, the score of molecular docking decreased less than in the RS-mutants with M231T mutation, which may be related to the higher induced expression level of *CYP51* in such mutants. According to our knowledge, this is the first report of M231T mutation on CYP51 protein in the DMI-resistant mutants, which needs further verification in the following work. Mutation K104E did not affect the binding of BcCYP51 and pyrisoxazole, and this amino acid position was also not conversed in different fungal species as shown in Figure 6A, suggesting it might be a linkage mutation with G476S. Similarly, a combined mutation at codons 103 and 157 in FgCYP51 was observed in *F. graminearum* metconazole-resistant mutants (Duan et al., 2018a).

In conclusion, baseline sensitivity using 110 *B. cinerea* isolates collected from nine provinces in China was established in this present study. As pyrisoxazole showed a high intrinsic activity on *B. cinerea* and no cross-resistance was found between pyrisoxazole and non-DMI fungicides, it indicated that pyrisoxazole can be used for better management of gray mold. Molecular docking models were used to predict how the mutations in the CYP51 binding pocket alter the binding of DMIs, suggesting that the G476S and M231T mutations combined with induced expression of the target gene appeared to mediate pyrisoxazole resistance of *B cinerea*. These results will be important for the control of the high-risk pathogen *B. cinerea* and may help in the development of more effective fungicides for disease management.

## Data Availability Statement

All datasets generated for this study are included in the article/Supplementary Material.

## Author Contributions

CZ and XL contributed to the conception and design of the study. CZ, MI, ML, ZL, and HG performed the experiments. CZ, XL, HD, and SZ analyzed the data. CZ wrote the first draft of the manuscript. XL supervised the study and revised the manuscript critically. All authors contributed to the revision of this manuscript.

## Conflict of Interest

The authors declare that the research was conducted in the absence of any commercial or financial relationships that could be construed as a potential conflict of interest.

## Footnotes

1 https://www.frac.info/

2 http://www.chinapesticide.org.cn

3 https://www.swissmodel.expasy.org/

## References

[B1] Albertini C. Thebaud G. Fournier E. Leroux P. (2002). Eburicol 14α-demethylase gene (CYP51) polymorphism and speciation in *Botrytis cinerea*. *Mycol. Res.* 106 1171–1178. 10.1017/s0953756202006561

[B2] Bardas G. A. Veloukas T. Koutita O. Karaoglanidis G. S. (2010). Multiple resistance of *Botrytis cinerea* from kiwifruit to SDHIs, QoIs and fungicides of other chemical groups. *Pest Manag. Sci.* 66 967–973. 10.1002/ps.1968 20730988

[B3] Birchmore R. J. Williams R. J. Russell P. E. Lagouarde P. (1996). A baseline for the sensitivity of *Botrytis cinerea* to pyrimethanil. *Brighton Crop Prot. Conf. Pests Dis.* 2 713–718.

[B4] Cai M. Lin D. Chen L. Bi Y. Xiao L. Liu X. L. (2015). M233I mutation in the β-tubulin of *Botrytis cinerea* confers resistance to zoxamide. *Sci. Rep. U.K.* 5:16881. 10.1038/srep16881 PMC4657022 26596626

[B5] Carter H. E. Fraaije B. A. West J. Kelly S. L. Mehl A. Shaw M. W. (2014). Alterations in the predicted regulatory and coding regions of the sterol 14α-demethylase gene (*CYP51*) confer decreased azole sensitivity in the oilseed rape pathogen *Pyrenopeziza brassicae*. *Mol. Plant Pathol.* 15, 513–522. 10.1111/mpp.12106 24298976 PMC6638911

[B6] Chen F. Lin D. Wang J. Li B. Duan H. Liu J. (2015). Heterologous expression of the *Monilinia fructicola* CYP51 (MfCYP51) gene in Pichia pastoris confirms the mode of action of the novel fungicide, SYP-Z048. *Front. Microbiol.* 6:457. 10.3389/fmicb.2015.00457 26042103 PMC4437033

[B7] Chen F. P. Fan J. R. Zhou T. Liu X. L. Liu J. L. Schnabel G. (2012). Baseline sensitivity of *Monilinia fructicola* from China to the DMI fungicide SYP-Z048 and analysis of DMI-resistant mutants. *Plant Dis.* 96 416–422. 10.1094/pdis-06-11-0495 30727143

[B8] Chen S. N. Luo C. X. Hu M. J. Schnabel G. (2016). Fitness and competitive ability of *Botrytis cinerea* isolates with resistance to multiple chemical classes of fungicides. *Phytopathology* 106 997–1005. 10.1094/phyto-02-16-0061-r 27161219

[B9] Chi M. H. Park S. Y. Lee Y. H. (2009). A quick and safe method for fungal DNA extraction. *Plant Pathol. J.* 25 108–111. 10.5423/ppj.2009.25.1.108

[B10] Cools H. J. Hawkins N. J. Fraaije B. A. (2013). Constraints on the evolution of azole resistance in plant pathogenic fungi. *Plant Pathol.* 62 36–42. 10.1111/ppa.12128

[B11] Duan Y. Li M. Zhao H. Lu F. Wang J. Zhou M. (2018a). Molecular and biological characteristics of laboratory metconazole-resistant mutants in *Fusarium graminearum*. *Pestic. Biochem. Phys.* 152 55–61. 10.1016/j.pestbp.2018.08.011 30497711

[B12] Duan Y. Li T. Xiao X. Wu J. Li S. Wang J. (2018b). Pharmacological characteristics of the novel fungicide pyrisoxazole against *Sclerotinia sclerotiorum*. *Pestic. Biochem. Phys.* 149 61–66. 10.1016/j.pestbp.2018.05.010 30033017

[B13] Elad Y. Williamson B. Tudzynski P. Delen N. (2007). “Botrytis spp. and diseases they cause in agricultural systems-an introduction,” in *Botrytis: Biology, Pathology and Control*, eds Elad Y. Williamson B. Tudzynski P. Delen N. (Dordrecht: Springer), 1–8. 10.1007/978-1-4020-2626-3_1

[B14] Fan F. Hamada M. S. Li N. Li G. Q. Luo C. X. (2017). Multiple fungicide resistance in *Botrytis cinerea* from greenhouse strawberries in hubei province. *China. Plant Dis.* 101 601–606. 10.1094/pdis-09-16-1227-re 30677353

[B15] Fan X. Zhang J. Yang L. Wu M. Chen W. Li G. (2015). Development of PCR-based assays for detecting and differentiating three species of *Botrytis* infecting broad bean. *Plant Dis.* 99 691–698. 10.1094/pdis-07-14-0701-re 30699675

[B16] Fernández-Ortuño D. Chen F. Schnabel G. (2012). Resistance to pyraclostrobin and boscalid in *Botrytis cinerea* isolates from strawberry fields in the carolinas. *Plant Dis.* 96 1198–1203. 10.1094/pdis-12-11-1049-re 30727059

[B17] Fujimura M. Banno S. Kamei M. Ishigami Y. Tsukada Y. (2016). Detection and monitoring of fungicide resistance in plant pathogens using pyrosequencing. *Jpn. J. Pestic. Sci.* 41, 78–87. 10.1584/jpestics.W15-36 27476087

[B18] Hahn M. (2014). The rising threat of fungicide resistance in plant pathogenic fungi: *Botrytis* as a case study. *J. Chem. Biol.* 7 133–141. 10.1007/s12154-014-0113-1 25320647 PMC4182335

[B19] Hayashi K. Schoonbeek H. De Waard M. A. (2002). Expression of the ABC transporter BcatrD from *Botrytis cinerea* reduces sensitivity to sterol demethylation inhibitor fungicides. *Pestic. Biochem. Phys.* 73 110–121. 10.1016/s0048-3575(02)00015-9

[B20] Hayashi K. Schoonbeek H. J. Sugiura H. De Waard M. A. (2001). Multidrug resistance in *Botrytis cinerea* associated with decreased accumulation of the azole fungicide oxpoconazole and increased transcription of the ABC transporter gene BcatrD. *Pestic. Biochem. Phys.* 70 168–179. 10.1006/pest.2001.2548

[B21] Jain A. N. (1996). Scoring noncovalent protein-ligand interactions: a continuous differentiable function tuned to compute binding affinities. *J. Comput. Aid. Mol. Des.* 10 427–440. 10.1007/bf00124474 8951652

[B22] Jain A. N. (2003). Surflex: fully automatic flexible molecular docking using a molecular similarity-based search engine. *J. Med. Chem.* 46 499–511. 10.1021/jm020406h 12570372

[B23] Kretschmer M. Hahn M. (2008). Fungicide resistance and genetic diversity of *Botrytis cinerea* isolates from a vineyard in Germany. *J. Plant Dis. Protec.* 115 214–219. 10.1007/bf03356266

[B24] Kretschmer M. Leroch M. Mosbach A. Walker A. S. Fillinger S. Mernke D. (2009). Fungicide-driven evolution and molecular basis of multidrug resistance in field populations of the grey mould fungus *Botrytis cinerea*. *PLoS Pathog.* 5:e1000696. 10.1371/journal.ppat.1000696 20019793 PMC2785876

[B25] Kumari S. Tayal P. Sharma E. Kapoor R. (2014). Analyses of genetic and pathogenic variability among *Botrytis cinerea* isolates. *Microbiol. Res.* 169 862–872. 10.1016/j.micres.2014.02.012 24767170

[B26] Leroux P. (2007). “Chemical control of *Botrytis* and its resistance to chemical fungicides,” in *Botrytis: Biology, Pathology and Control*, eds Elad Y. Williamson B. Tudzynski P. Delen N. (Dordrecht: Springer), 195–222. 10.1007/978-1-4020-2626-3_12

[B27] Leroux P. Walker A. S. (2013). Activity of fungicides and modulators of membrane drug transporters in field strains of *Botrytis cinerea* displaying multidrug resistance. *Eur. J. Plant Pathol.* 135 683–693. 10.1007/s10658-012-0105-3

[B28] Lichtemberg P. S. Luo Y. Morales R. G. Muehlmann-Fischer J. M. Michailides T. J. May De Mio L. L. (2017). The point mutation G461S in the MfCYP51 gene is associated with tebuconazole resistance in *Monilinia fructicola* populations in Brazil. *Phytopathology* 107 1507–1514. 10.1094/phyto-02-17-0050-r 28697663

[B29] Liu J. L. Si N. G. Chen L. Zhang D. M. Zhang Z. J. (2004). Biological activity against tomato leaf mold and application of a novel fungicide, SYP-Z048 (III). *Chin. J. Pestic.* 43 103–105.

[B30] Liu S. Che Z. Chen G. (2016). Multiple-fungicide resistance to carbendazim, diethofencarb, procymidone, and pyrimethanil in field isolates of *Botrytis cinerea* from tomato in henan province, China. *Crop Prot.* 84 56–61. 10.1016/j.cropro.2016.02.012

[B31] Livak K. J. Schmittgen T. D. (2001). Analysis of relative gene expression data using real-time quantitative PCR and the 2-ΔΔCT method. *Methods* 25 402–408. 10.1006/meth.2001.1262 11846609

[B32] Ma Z. Michailides T. J. (2005). Advances in understanding molecular mechanisms of fungicide resistance and molecular detection of resistant genotypes in phytopathogenic fungi. *Crop Prot.* 24 853–863. 10.1016/j.cropro.2005.01.011

[B33] Mair W. Lopez-Ruiz F. Stammler G. Clark W. Burnett F. Hollomon D. (2016). Proposal for a unified nomenclature for target-site mutations associated with resistance to fungicides. *Pest Manag. Sci.* 72 1449–1459. 10.1002/ps.4301 27148866 PMC5094580

[B34] Markoglou A. N. Ziogas B. N. (2002). SBI-fungicides: fungicidal effectiveness and resistance in “*Botrytis cinerea*”. *Phytopathol. Mediterr.* 41 120–130.

[B35] Moyano C. Gómez V. Melgarejo P. (2004). Resistance to pyrimethanil and other fungicides in *Botrytis cinerea* populations collected on vegetable crops in Spain. *J. Phytopathol.* 152 484–490. 10.1111/j.1439-0434.2004.00880.x

[B36] Myresiotis C. K. Karaoglanidis G. S. Tzavella-Klonari K. (2007). Resistance of *Botrytis cinerea* isolates from vegetable crops to anilinopyrimidine, phenylpyrrole, hydroxyanilide, benzimidazole, and dicarboximide fungicides. *Plant Dis.* 91 407–413. 10.1094/pdis-91-4-0407 30781182

[B37] Qian H. Du J. Chi M. Sun X. Liang W. Huang J. (2018). The Y137H mutation in the cytochrome P450 FgCYP51B protein confers reduced sensitivity to tebuconazole in *Fusarium graminearum*. *Pest Manag. Sci.* 74 1472–1477. 10.1002/ps.4837 29274114

[B38] Rosslenbroich H. J. Stuebler D. (2000). *Botrytis cinerea*-history of chemical control and novel fungicides for its management. *Crop Prot.* 19 557–561. 10.1016/s0261-2194(00)00072-7

[B39] Russell P. E. (2004). *Sensitivity Baseline In Fungicide Resistance Research, And Management.* Brussels: FRAC Press.

[B40] Samuel S. Papayiannis L. C. Leroch M. Veloukas T. Hahn M. Karaoglanidis G. S. (2011). Evaluation of the incidence of the G143A mutation and cytb intron presence in the cytochrome bc-1 gene conferring QoI resistance in *Botrytis cinerea* populations from several hosts. *Pest Manag. Sci.* 67 1029–1036. 10.1002/ps.2226 21702077

[B41] Shao J. X. Zhou X. F. Zhang J. X. (2006). Field experimental results in controlling tomato gray mold with SYP-Z048 25% EC. *Agrochemicals* 45 488–490.

[B42] Si N. G. Zhang Z. J. Liu J. L. Li Z. N. Zhang D. M. Chen L. (2004a). Biological activity and application of a novel fungicide: SYP-Z048. *Chin. J. Pestic.* 43 16–18.

[B43] Si N. G. Zhang Z. J. Liu J. L. Li Z. N. Zhan D. M. Chen L. (2004b). Biological activity and application of a novel fungicide, SYP-Z048 (II). *Chin. J. Pestic.* 43 61–63.

[B44] Sionov E. Chang Y. C. Garraffo H. M. Dolan M. A. Ghannoum M. A. Kwon-Chung K. J. (2012). Identification of a *Cryptococcus neoformans* cytochrome P450 lanosterol 14α-demethylase (Erg11) residue critical for differential susceptibility between fluconazole/voriconazole and itraconazole/posaconazole. *Antimicrob. Agents Chem.* 56 1162–1169. 10.1128/aac.05502-11 22155829 PMC3294891

[B45] Song Y. Xu D. Lu H. He L. Chen L. Shao J. (2016). Baseline sensitivity and efficacy of the sterol biosynthesis inhibitor triflumizole against *Botrytis cinerea*. *Austr. Plant Pathol.* 45 65–72. 10.1007/s13313-015-0384-1

[B46] Williamson B. Tudzynski B. Tudzynski P. van Kan J. A. (2007). *Botrytis cinerea*: the cause of grey mould disease. *Mol. Plant Pathol.* 8 561–580. 10.1111/j.1364-3703.2007.00417.x 20507522

[B47] Yin W. X. Adnan M. Shang Y. Lin Y. Luo C. X. (2018). Sensitivity of *Botrytis cinerea* from nectarine/cherry in China to six fungicides and characterization of resistant isolates. *Plant Dis.* 102 578. 10.1094/PDIS-02-18-0244-RE 30299208

[B48] Zhang C. Diao Y. Wang W. Hao J. Imran M. Duan H. (2017). Assessing the risk for resistance and elucidating the genetics of *Colletotrichum truncatum* that is only sensitive to some DMI fungicides. *Front. Microbiol.* 8:1779 10.3389/fmicb.2015.001779 PMC5609536 28970822

[B49] Zhang C. Q. Hu J. L. Wei F. L. Zhu G. N. (2009). Evolution of resistance to different classes of fungicides in *Botrytis cinerea* from greenhouse vegetables in eastern China. *Phytoparasitica* 37 351–359. 10.1007/s12600-009-0050-7

[B50] Zhang Y. Zhou Q. Tian P. Li Y. Duan G. Li D. (2019). Induced expression of CYP51 associated with difenoconazole resistance in the pathogenic Alternaria sect. on potato in China. *Pest Manag. Sci.* 76 1751–1760. 10.1002/ps.5699 31785067

[B51] Zhu H. Huang C. T. Ji M. S. (2016). Baseline sensitivity and control efficacy of pyrisoxazole against *Botrytis cinerea*. *Eur. J. Plant Pathol.* 146 315–323. 10.1007/s10658-016-0917-7

[B52] Zwiers L. H. Stergiopoulos L. Van Nistelrooy J. G. M. De Waard M. A. (2002). ABC transporters and azole susceptibility in laboratory strains of the wheat pathogen *Mycosphaerella graminicola*. *Antimicrob. Agents Chemother.* 46 3900–3906. 10.1128/aac.46.12.3900-3906.2002 12435694 PMC132773

