# Supplementary material for: Two Point Mutations on CYP51 Combined With Induced Expression of the Target Gene Appeared to Mediate Pyrisoxazole Resistance in Botrytis cinerea
Source: Front Microbiol. 2020 Jun 30;11:1396. doi: 10.3389/fmicb.2020.01396 (PMC7340008; doi:10.3389/fmicb.2020.01396)
Supplement: Supplementary file 2 [file Data_Sheet_1.docx]

**Table S1. Background data regarding the 110 *Botrytis cinerea* isolates in China.**

| **Location (province, city)** | **Number of isolates** | **EC_50_ range (μg/ml)** | **Mean EC_50_ (μg/ml)** |
| --- | --- | --- | --- |
| Fujian, Ningde | 9 | 0.021-0.166 | 0.051 |
| Liaoning, Dandong | 15 | 0.025-0.036 | 0.027 |
| Inner Mongolia, Chifeng | 12 | 0.020-0.091 | 0.058 |
| Shanghai, Baoshan | 11 | 0.049-0.082 | 0.060 |
| Shandong, Yantai | 14 | 0.055-0.078 | 0.056 |
| Henan, Xinxiang | 12 | 0.059-0.130 | 0.083 |
| Beijing, Daxing | 12 | 0.055-0.147 | 0.089 |
| Tianjin, Wuqing | 10 | 0.041-0.051 | 0.045 |
| Jiangsu, Suqian | 15 | 0.033-0.084 | 0.051 |
| Total | 110 | 0.020-0.166 | 0.057 |

**Table S2. Concentrations used to determine the sensitivity of *Botrytis cinerea* field isolate and resistant mutants to various fungicides.**

| **Fungicide** | **Fungicide concentration (μg/ml) in agar medium** |
| --- | --- |
| Pyrisoxazole | 0, 0.01, 0.03, 0.05, 0.1, 0.2. 0.5 for wide-type parental isolate  0, 0.05, 0.1, 0.2, 0.5, 1.0, 2.0 for resistant mutants^a^ |
| Tebuconazole | 0, 0.05, 0.1, 0.2, 0.5, 1.0 for wide-type parental isolate  0, 0.1, 0.5, 1.0, 5.0, 10.0 for resistant mutants |
| Prochloraz | 0, 0.02, 0.05, 0.1, 0.2, 0.5, 1.0 for wide-type parental isolate  0, 0.1, 0.2, 0.5, 1.0, 5.0, 10.0 for resistant mutants |
| Iprodione | 0, 0.2, 0.5, 1.5, 2.0, 4.0 |
| Procymidone | 0, 0.1. 0.25, 0.3, 0.4, 1.0 |
| Dithofencarb | 0, 0.05, 0.1, 0.2, 0.5, 1.0, 3.0 |
| Fluazinam | 0, 0.003, 0.005, 0.01, 0.03, 0.05, 0.1 |
| Pyrimethanil | 0, 0.05, 0.1, 0.15, 0.20, 0.40 |
| Fludioxonil | 0, 0.001, 0.005, 0.01, 0.05, 0.1, 0.5 |

^a^The mutants were selected in the laboratory by exposing them to pyrisoxazole.

**Table S2. Concentrations used to determine the sensitivity of** ***Botrytis cinerea* field isolate and resistant mutants to various fungicides.**

| **Fungicide** | **Fungicide concentration (μg/ml) in agar medium** |
| --- | --- |
| Pyrisoxazole | 0, 0.01, 0.03, 0.05, 0.1, 0.2. 0.5 for wide-type parental isolate  0, 0.05, 0.1, 0.2, 0.5, 1.0, 2.0 for resistant mutants^a^ |
| Tebuconazole | 0, 0.05, 0.1, 0.2, 0.5, 1.0 for wide-type parental isolate  0, 0.1, 0.5, 1.0, 5.0, 10.0 for resistant mutants |
| Prochloraz | 0, 0.02, 0.05, 0.1, 0.2, 0.5, 1.0 for wide-type parental isolate  0, 0.1, 0.2, 0.5, 1.0, 5.0, 10.0 for resistant mutants |
| Iprodione | 0, 0.2, 0.5, 1.5, 2.0, 4.0 |
| Procymidone | 0, 0.1. 0.25, 0.3, 0.4, 1.0 |
| Dithofencarb | 0, 0.05, 0.1, 0.2, 0.5, 1.0, 3.0 |
| Fluazinam | 0, 0.003, 0.005, 0.01, 0.03, 0.05, 0.1 |
| Pyrimethanil | 0, 0.05, 0.1, 0.15, 0.20, 0.40 |

^a^The mutants were selected in the laboratory by exposing them to pyrisoxazole.
